# Supplementary material for: Interpretable adenylation domain specificity prediction using protein language models
Source: bioRxiv. 2025 Jan 18:2025.01.13.632878. Preprint. [Version 1] doi: 10.1101/2025.01.13.632878 (PMC11761653; doi:10.1101/2025.01.13.632878)
Supplement: Supplement 1 [file NIHPP2025.01.13.632878v1-supplement-1.pdf]

bioRxiv preprint doi: https://doi.org/10.1101/2025.01.13.632878; this version posted January 18, 2025. The copyright holder for this preprint (which was not certified by peer review) is the author/funder, who has granted bioRxiv a license to display the preprint in perpetuity. It is made available under aCC-BY 4.0 International license.

Name	BGC	Seq2Hybrid Tanimoto	Prism 4 Tanimoto
Rakicidin B	BGC0001327	1	0.211
Micromonolactam	NA	1	0.747
Hapalosin	BGC0001467	1	0.744
JBIR-06	BGC0001918	1	0.693
Neoantimycin	BGC0001695	1	0.678
Cystothiazole A	BGC0000982	1	0.614
Kasumigamide	BGC0001630	1	0.472
Incednine	BGC0000078	1	0.306
Pyridomycin	BGC0001039	1	0.384
Rufomycin	BGC0001763	1	0.27
Ilamycins	BGC0001620	1	0.27
Splenocin C	BGC0001216	1	0.532
Eponemycin	BGC0000345	1	0.185
Nostopeptolide	BGC0001028	0.924	0.752
Skyllamycin	BGC0000429	0.852	0.56
Leucinostatin A	BGC0001358	0.802	0.12
Paenilipoheptin	BGC0001728	0.796	0.463
Althiomycin	BGC0000955	0.791	0.252
Tubulysin	BGC0001344	0.762	0.364
Cryptomaldamide	BGC0001560	0.758	0.393
Bacillomycin D	BGC0001090	0.757	0.238
Salinilactam	BGC0000142	0.753	0.51
Lobosamide	BGC0001303	0.747	0.505
Myxochromide A	BGC0001425	0.739	0.41
Myxochromide C	BGC0001423	0.736	0.48

Supplementary Table S1. Seq2Hybrid vs PRISM 4 performance at recovering NRP-PK hybrid molecules at or above a Tanimoto similarity threshold of 0.7 in either the training or test set.

Strains
Streptomyces atratus NBRC 3897
Streptomyces pyridomyceticus NRRL B-2517
Streptomyces orinoci NBRC 13466
Micromonospora chalcea NRRL B-2672
Streptomyces alboflavus strain MDJK44
Streptomyces hygroscopicus NRRL B-1477
Streptomyces kasugaensis NBRC 13851
Streptomyces violascens NRRL B-2700

Supplementary Table S2. Strains used in this study for which mass spectrometry data was obtained.

bioRxiv preprint doi: https://doi.org/10.1101/2025.01.13.632878; this version posted January 18, 2025. The copyright holder for this preprint (which was not certified by peer review) is the author/funder, who has granted bioRxiv a license to display the preprint in perpetuity. It is made available under aCC-BY 4.0 International license.

Substrate	SMILES
Arg	C(CC(C(=O)O)N)CN=C(N)N
His	C1=C(NC=N1)CC(C(=O)O)N
Lys	C(CCN)CC(C(=O)O)N
Asp	C(C(C(=O)O)N)C(=O)O
Glu	C(CC(=O)O)C(C(=O)O)N
Ser	C(C(C(=O)O)N)O
Thr	CC(C(C(=O)O)N)O
Asn	C(C(C(=O)O)N)C(=O)N
Gln	C(CC(=O)N)C(C(=O)O)N
Cys	C(C(C(=O)O)N)S
Gly	C(C(=O)O)N
Pro	C1CC(NC1)C(=O)O
Ala	CC(C(=O)O)N
Val	CC(C)C(C(=O)O)N
Ile	CCC(C)C(C(=O)O)N
Leu	CC(C)CC(C(=O)O)N
Met	CSCCC(C(=O)O)N
Phe	C1=CC=C(C=C1)CC(C(=O)O)N
Tyr	C1=CC(=CC=C1CC(C(=O)O)N)O
Trp	C1=CC=C2C(=C1)C(=CN2)CC(C(=O)O)N
Orn	C(CC(C(=O)O)N)CN
5hfOrn	C(CC(C(=O)O)N)CN(C=O)O
5hOrn	C(CC(C(=O)O)N)CNO
4-MePro	CC1CC(NC1)C(=O)O
Bht	C1=CC(=CC=C1C(C(C(=O)O)N)O)O
Hty	C1=CC(=CC=C1CCC(C(=O)O)N)O
MeDap	CC(C(C(=O)O)N)N
MeAsp	CC(C(C(=O)O)N)C(=O)O
HyAsp	OC(C(C(=O)O)N)C(=O)O
CAM	OC(=O)C(N)C1CCNC(=N)N1
hEnd	C1C(NC(N1)=N)C(C(C(=O)O)N)O
Dab	OC(=O)C(N)CCN
l-2,3-DAP	OC(=O)C(N)CN
Aad	C(CC(C(=O)O)N)CC(=O)O
Dhpg	C1=C(C=C(C=C1O)O)C(C(=O)O)N
Hpg	C1=CC(=CC=C1C(C(=O)O)N)O
Dhb	C1=CC(=C(C(=C1)O)O)C(=O)O
Beta-ala	C(CN)C(=O)O
Hyv-d	CC(CO)C(C(=O)O)N
Pip	C1CCNC(C1)C(=O)O
Dht	CC(=C(C(=O)O)N)O

Supplementary Table S3. Substrates used in this study to train MASPR and their corresponding SMILES representations.
